# Superior mesenteric artery syndrome

**DOI:** 10.1002/ccr3.3118

**Published:** 2020-07-16

**Authors:** Bipin Karki, Bishika Pun, Amit Shrestha, Pramesh Sunder Shrestha

**Affiliations:** ^1^ Department of Critical Care Medicine Om Hospital and Research Center Kathmandu Nepal; ^2^ Department of Radiology Om Hospital and Research Center Kathmandu Nepal; ^3^ Department of Radiology Nepal Medical College and Teaching Hospital Kathmandu Nepal; ^4^ Department of Anaesthesiology Tribhuvan University Teaching Hospital Kathmandu Nepal

**Keywords:** aorto‐mesenteric angle, aorto‐mesenteric distance, duodenal obstruction, Superior mesenteric artery syndrome

## Abstract

Superior mesenteric artery (SMA) syndrome, though rare, should be considered in patients with duodenal obstruction with no other causes. History of recent weight loss and imaging modalities help in the diagnosis. Conservative management can be tried before going for surgery.

## Graphical abstract

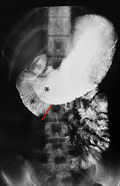

A 21‐year‐old female presented with history of vague abdominal discomfort, vomiting, and weight loss for 6 months. The vomiting had gotten more frequent in the last 2 months, a few hours following almost every meal. She had lost about 8 kg body weight in 6 months. Her body mass index was 17.36 kg/m^2^. Following an unremarkable ultrasound of the abdomen and upper gastrointestinal endoscopy, a barium meal was obtained which showed an abrupt cutoff at the third part of duodenum with proximal distention (Figure 1). A contrast‐enhanced computerized tomography (CECT) showed an aorto‐mesenteric angle (AO) of 20° (Figure 2) and the aorto‐mesenteric distance (AO) of 2.9 mm (Figure 3). Superior mesenteric artery (SMA) syndrome was diagnosed. The patient was kept under conservative management. High‐calorie, low‐volume diet and prokinetic agents were prescribed via a naso‐jejunal tube. She was symptomatically better and was gaining weight. Oral feeding was encouraged, and naso‐jejunal tube was removed by the third week. However, she was lost to follow‐up after 3 months.

**FIGURE 1:**
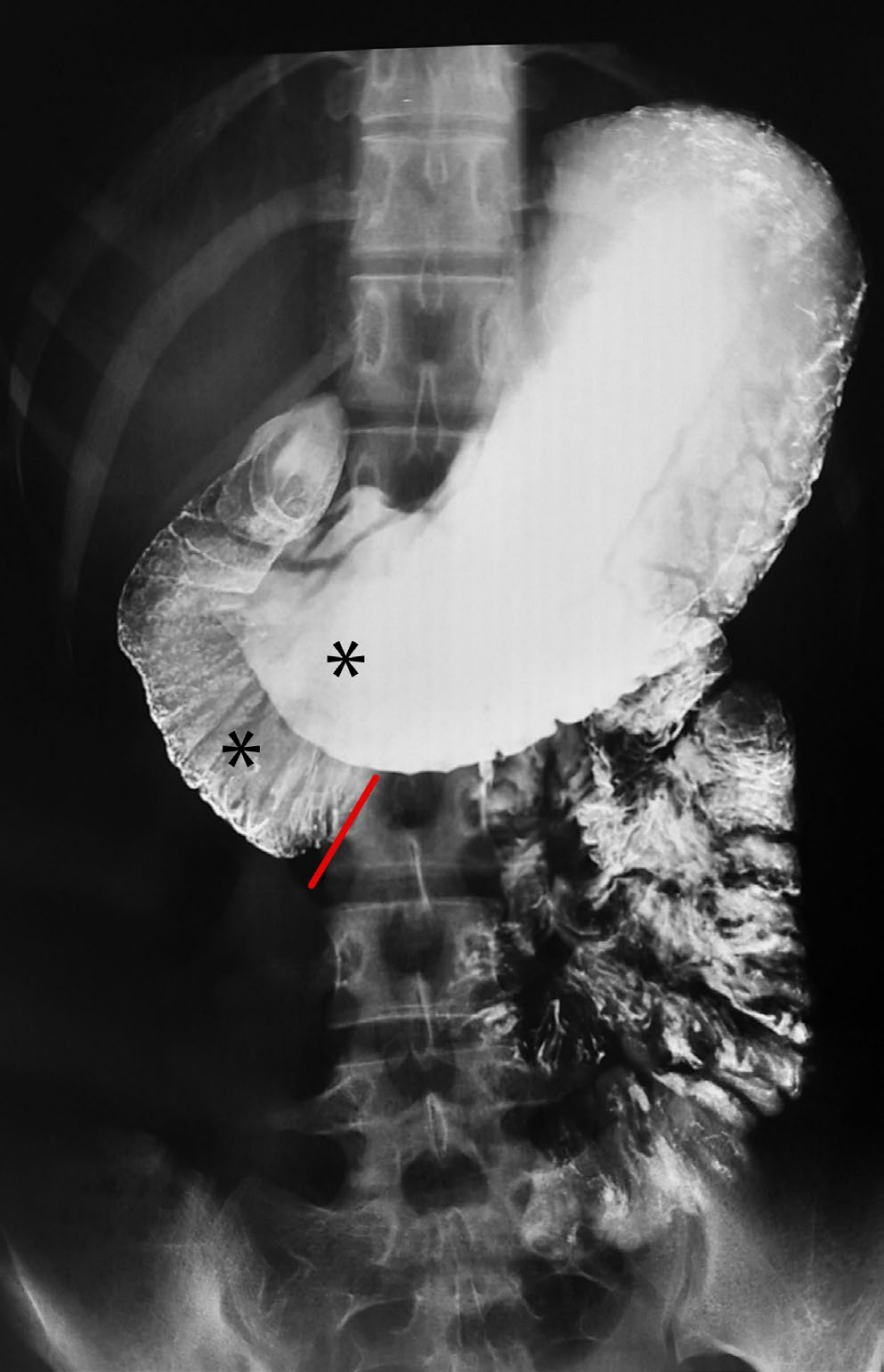
Barium meal showing distended stomach, first and second part of duodenum (*) with abrupt cutoff at third part of duodenum (red line)

**FIGURE 2:**
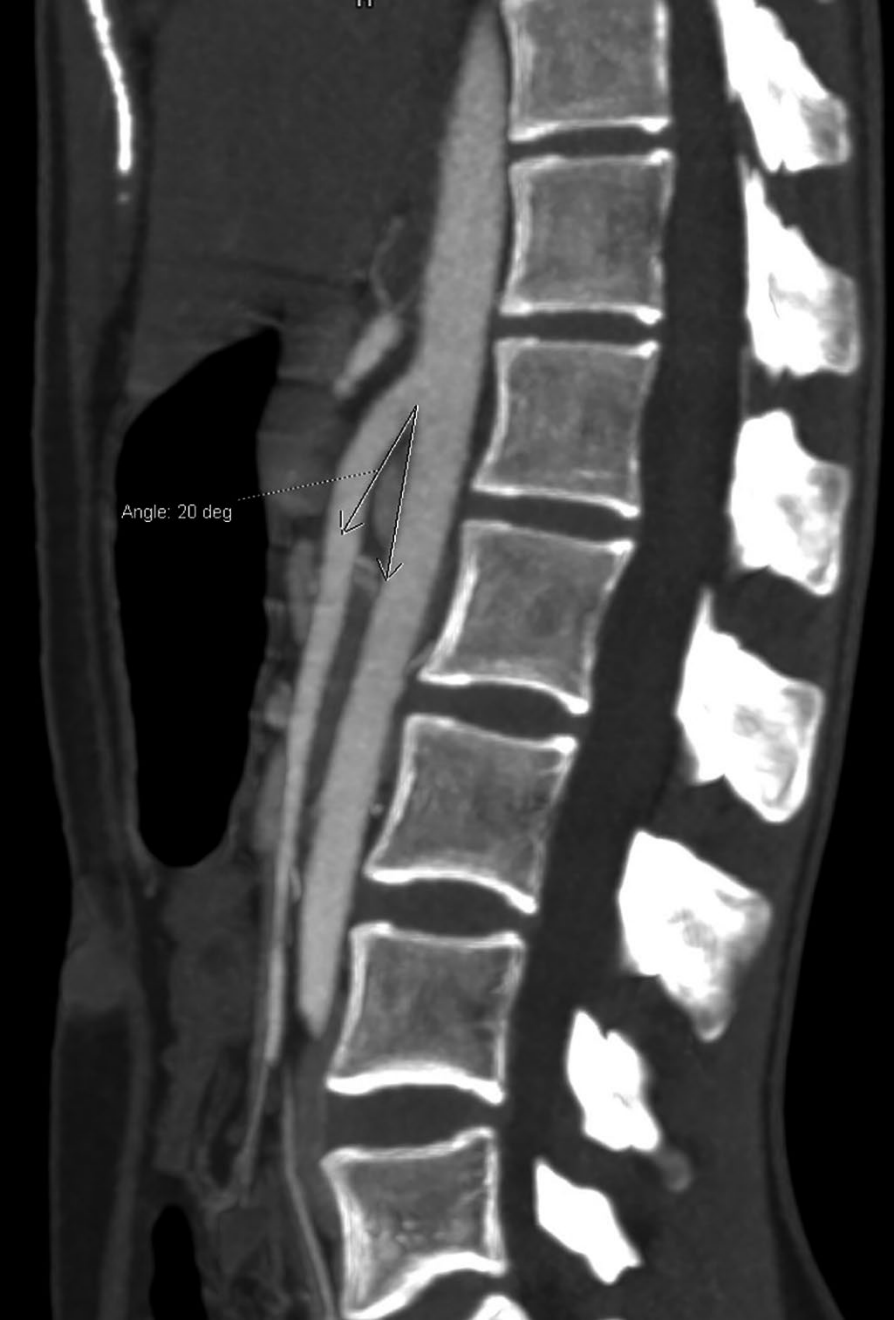
Sagittal section of CECT abdomen showing a narrowed AO angle of 20°

**FIGURE 3:**
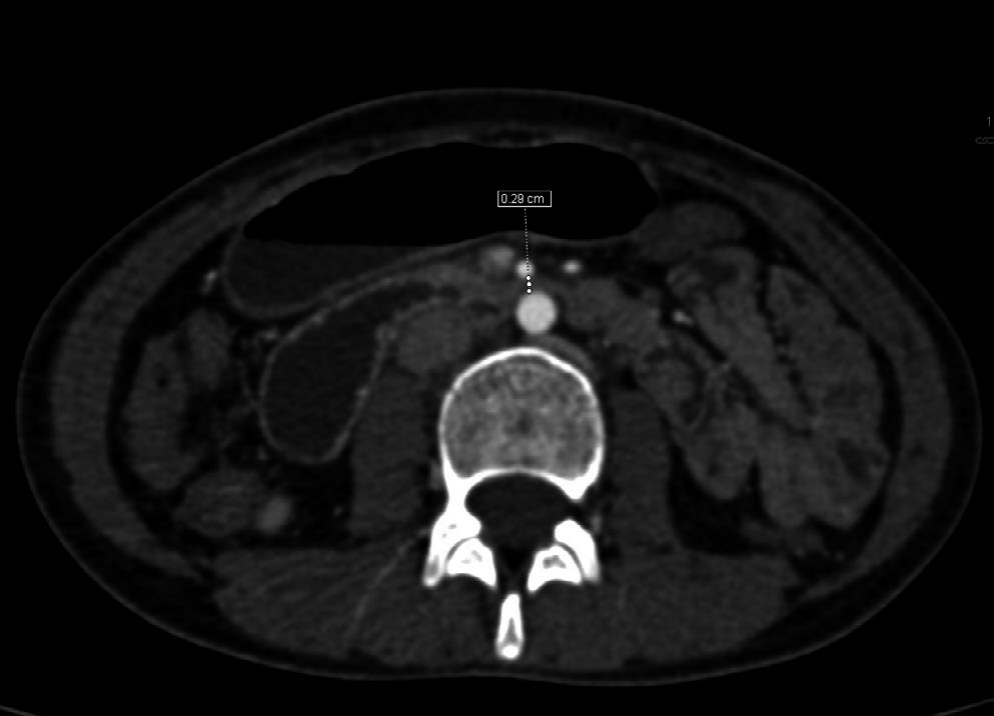
Axial section of CECT abdomen showing a decreased AO distance of 2.9 mm

Superior mesenteric artery syndrome is considered one of the rare causes of duodenal obstruction. Reduced AO angle (<22‐28°) and AO distance (2‐8 mm) are highly suggestive.^1^ Though conservative medical management is initially considered, surgery is required in many cases.^2^

## CONFLICT OF INTEREST

None declared.

## AUTHOR CONTRIBUTIONS

BP and BK: involved in initial drafting of manuscript; AS and PSS: involved in patient care and review of the images; all authors reviewed and finalized the manuscript.

## ETHICAL APPROVAL

Not applicable.

## Consent statement

Published with written consent of the patient.
